# Bactericidal antibiotics induce programmed metabolic toxicity

**DOI:** 10.15698/mic2016.04.493

**Published:** 2016-03-09

**Authors:** Aislinn D. Rowan, Damien J. Cabral, Peter Belenky

**Affiliations:** 1 Department of Molecular Microbiology and Immunology, Brown University, 171 Meeting Street, Providence, RI 02912.

**Keywords:** antibiotics, reactive oxygen species (ROS), metabolism, antibiotic resistance and tolerance

## Abstract

The misuse of antibiotics has led to the development and spread of antibiotic resistance in clinically important pathogens. These resistant infections are having a significant impact on treatment outcomes and contribute to approximately 25,000 deaths in the U.S. annually. If additional therapeutic options are not identified, the number of annual deaths is predicted to rise to 317,000 in North America and 10,000,000 worldwide by 2050. Identifying therapeutic methodologies that utilize our antibiotic arsenal more effectively is one potential way to extend the useful lifespan of our current antibiotics. Recent studies have indicated that modulating metabolic activity is one possible strategy that can impact the efficacy of antibiotic therapy. In this review, we will address recent advances in our knowledge about the impacts of bacterial metabolism on antibiotic effectiveness and the impacts of antibiotics on bacterial metabolism. We will particularly focus on two studies, Lobritz, *et al.* (PNAS, 112(27): 8173-8180) and Belenky *et al.* (Cell Reports, 13(5): 968-980) that together demonstrate that bactericidal antibiotics induce metabolic perturbations that are linked to and required for bactericidal antibiotic toxicity.

These two papers build on a significant body of evidence that suggests that reduced metabolic activity leads to an antibiotic-tolerant state and that elevated metabolic activity contributes to bactericidal antibiotic toxicity in susceptible bacteria (Figure 1). For example, *Mycobacterium tuberculosis* can become antibiotic-tolerant by entering a metabolically quiescent, slow-growing state. This is accomplished by shunting carbon flux away from the tricarboxylic acid (TCA) cycle and into storage as triacylglycerol instead. If this pathway is inhibited, *M. tuberculosis* remains susceptible to a variety of antibiotics when placed under stress, providing a potential therapeutic target to increase the efficacy of available drugs. The TCA cycle is also an important determinant of antibiotic tolerance in the opportunistic pathogen *Staphylococcus epidermidis*. In *S. epidermidis*, disabling the TCA cycle has a protective effect against beta-lactam antibiotics due to suppression of reactive oxygen species (ROS) generation during antibiotic exposure, as well as increased tolerance of oxidative stress. TCA cycle defects are also common in clinical isolates of *S. epidermidis*, suggesting that such mutations may be a common antibiotic defense. In addition, in *Staphylococcus aureus* and *Escherichia coli*, antibiotic-tolerant persister and biofilm cells can have reduced proton motive force (PMF), leading to inhibited uptake of aminoglycoside antibiotics (Figure 1). This tolerance can be reversed by the addition of metabolism-stimulating sugars that boost PMF and the uptake of antibiotics.

**Figure 1:**
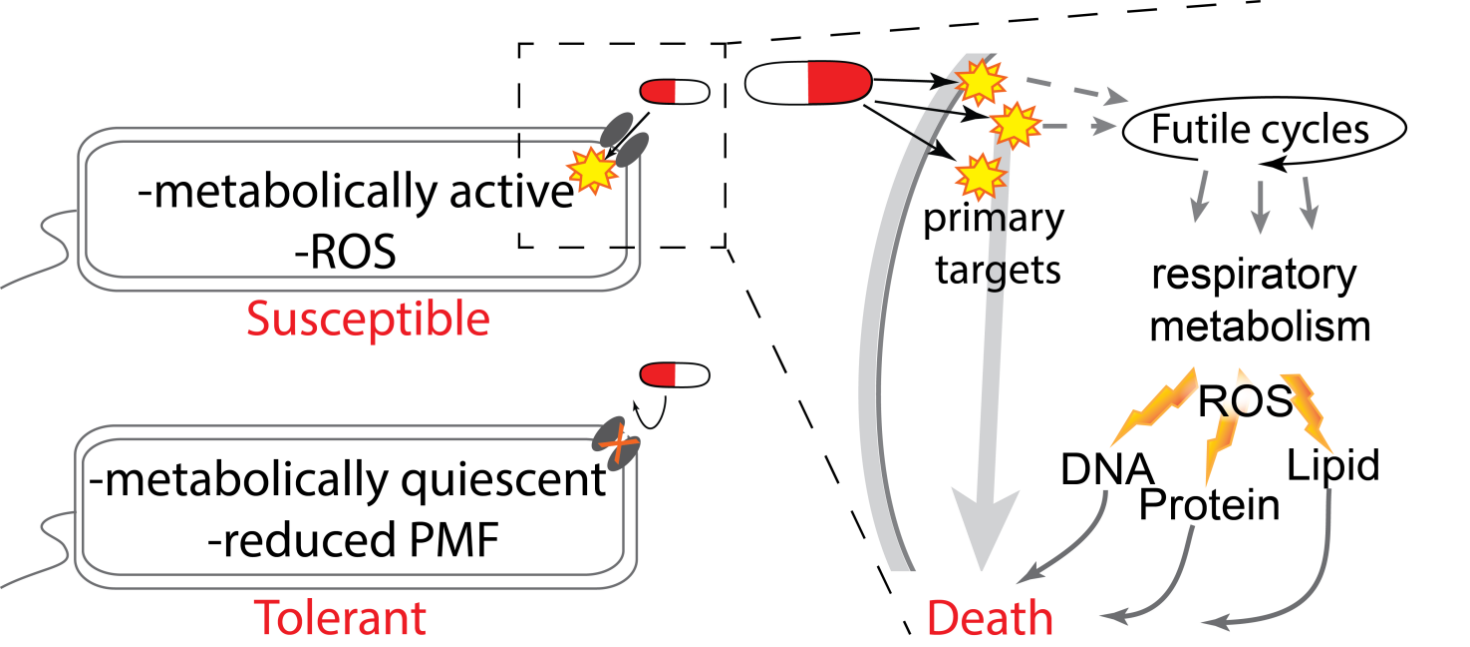
FIGURE 1: A model relating the impact of respiratory metabolism on bactericidal toxicity. Metabolically quiescent cells are tolerant to antibiotics whereas metabolically active bacteria are more susceptible. In metabolically active cells bactericidal antibiotics interact with their primary targets to directly induce toxicity. In addition to direct toxicity this initial target dependent damage also leads to the elevation of respiratory activity through the induction of futile metabolic cycles and other mechanisms. Elevated redox metabolism leads to elevated oxygen consumption and induction of ROS production as both a metabolic byproduct and as a result of disordered respiratory activity. ROS and other oxidants then damage cellar components such as DNA, protein and lipids and thereby contribute to bacterial death.

A growing compendium of evidence suggests that in non-tolerant bacteria, elevated central metabolism contributes to bactericidal antibiotic toxicity through the production of reactive oxidants and other damaging molecules. At low therapeutically-relevant concentrations, drug-target interactions of bacteriostatic antibiotics induce ROS as the principal cause of antibiotic toxicity under oxygenated conditions. At higher concentrations of antibiotics, ROS and other damaging reactive species appear to play a lesser role in toxicity. The link between ROS and antibiotic toxicity has been recently disputed, but in general a preponderance of evidence suggests that the induction of ROS and metabolic activity are inexorably linked to bacterial death.

Initial evidence about this metabolic link came from a 2013 article by Brynildsen *et al.*, which utilized *in silico* genome scale metabolic models to identify perturbations that increase intracellular production of endogenous ROS species. In particular, they found that perturbations to the production or usage of ATP were associated with predicted increases in ROS. Chiefly, targeting glycolysis, the TCA cycle, and aerobic respiration led to endogenous ROS production and susceptibility to antibiotic-induced oxidative stress. Subsequent work identified experimentally-tractable futile cycling reactions that decrease net intracellular ATP levels. Furthermore, actively cycling strains were significantly more susceptible to oxidative stress and certain antibiotics (aminoglycosides). This suggests that this increase in sensitivity is attributable to increased damage and/or impaired repair mechanisms caused by perturbations in ROS. Along with other recent studies, this has forcefully demonstrated that artificially inducing oxidative metabolism can impact antibiotic susceptibility. Under natural conditions, metabolically-costly futile cycles also contribute to bactericidal efficacy. Cho *et al*. demonstrated that the bactericidal activity of beta-lactam antibiotics is due, in part, to the induction of a futile cycle between peptidoglycan cell wall synthesis and degradation. This is a metabolically-costly process that likely contributes considerably to ATP consumption, as seen in previous futile cycle studies. The total body of previous work suggests that metabolic activity contributing to ROS production is an integral part of bactericidal toxicity.

The two papers at the heart of this microreview, by Lobritz *et al.* (PNAS, 112(27): 8173-8180) and Belenky *et al.* (Cell Reports, 13(5): 968-980) expand on this foundation of work by testing the impacts of bactericidal antibiotics on respiratory metabolism and the toxic consequences of this elevated metabolic activity respectively. Lobritz *et al.* utilized a novel high-throughput microplate-based method of analyzing bacterial oxygen consumption (using the Seahorse Extracellular Flux Analyzer). They found that bactericidal antibiotics induced a rapid induction in oxygen consumption indicative of elevated respiration (Figure 1). On the other hand, the same assay found that treatment with bacteriostatic antibiotics rapidly and permanently eliminated essentially all respiratory activity. They also found that inhibiting respiration eliminated bactericidal activity, whereas increasing respiration led to boosted toxicity. This indicates that induction of respiration is essential for bactericidal antibiotic efficacy. Finally, and most importantly, this work identified that many bacteriostatic drugs are antagonistic to bactericidal drugs when used in combinatorial therapy, because the bacteriostatic-dependent inhibition of respiration supersedes the bactericidal respiration-based benefit. The impact of this observation is that many currently utilized empirical therapeutic combinations are detrimental because they may lead to direct inhibition of an essential metabolic burst.

Belenky *et al.* continued the study of antibiotic-induced metabolic activity by conducting a total metabolomic profile of antibiotic-treated *E. coli*. This work found that bactericidal antibiotics - aminoglycosides, fluoroquinolones and beta-lactams - induce a common initial set of metabolic perturbations. These changes included an induction of central carbon metabolites, exemplified by dramatically elevated TCA cycle intermediates, as well as raised levels of glutathione, which are suggestive of an active antioxidant response. These data are consistent with the bacteriotoxic metabolic burst hypothesis proposed and tested in Lobritz *et al.* In addition to the TCA cycle and glutathione signatures, Belenky *et al.* also found metabolic signatures suggestive of nucleotide damage and perturbed lipid homeostasis. This observation led them to test potential end-target consequences of metabolically-induced oxidative stress. They found that bactericidal antibiotics lead to higher levels of malondialdehyde adducts, protein carbonylation, and 8-oxo-guanine, indicative of lipid, protein, and nucleotide oxidation respectively (Figure 1). Finally using a fluorescence-based protein assay, they showed that antibiotic treatment induced double-strand DNA breaks likely due to oxidative damage.

When taken together, the two papers make a very convincing argument about the importance of a metabolic burst for the damage of bacterial cells and eventual toxicity during bactericidal treatment. This perspective comes at a very critical time, when antibiotic efficacy is being increasingly compromised by rising rates of resistance. Modulating metabolic activity may provide important therapeutic methodologies to extend the useful life span of our current antibiotics. In addition, the metabolic signature of this respiratory burst could be used as a potent readout to aid in the development of new drugs and for screening of antibiotic resistance in laboratory and clinical settings. In general, the developing perspective that antibiotic-induced death is not a single, one-step response but rather a programmed set of discernable processes is a wonderful change. This new perspective opens up a large set of cellular processes that can be modulated and utilized to improve therapy. Before this appreciation, antibiotic research was very much limited to drug-target interactions, while now the entire cell is open for business.

